# The Role of Circadian Clock Genes in Critical Illness: The Potential Role of Translational Clock Gene Therapies for Targeting Inflammation, Mitochondrial Function, and Muscle Mass in Intensive Care

**DOI:** 10.1177/07487304221092727

**Published:** 2022-06-07

**Authors:** Joanna Poole, David Ray

**Affiliations:** * Anaesthetics and Critical Care, Gloucestershire Royal Hospital, Gloucestershire Hospitals NHS Foundation Trust, Gloucester, UK; † NIHR Oxford Biomedical Research Centre, John Radcliffe Hospital, Oxford, UK; ‡ Oxford Centre for Diabetes, Endocrinology and Metabolism, University of Oxford, Oxford, UK

**Keywords:** circadian, chronotherapy, intensive care, critical care, inflammation, acute respiratory distress syndrome, ventilator-associated lung injury, therapy, brain injury, multiorgan failure, critical illness, mitochondria, inflammasome, immunomodulation, cytokines, acute kidney injury, epigenetic

## Abstract

The Earth’s 24-h planetary rotation, with predictable light and heat cycles, has driven profound evolutionary adaptation, with prominent impacts on physiological mechanisms important for surviving critical illness. Pathways of interest include inflammation, mitochondrial function, energy metabolism, hypoxic signaling, apoptosis, and defenses against reactive oxygen species. Regulation of these by the cellular circadian clock (BMAL-1 and its network) has an important influence on pulmonary inflammation; ventilator-associated lung injury; septic shock; brain injury, including vasospasm; and overall mortality in both animals and humans. Whether it is cytokines, the inflammasome, or mitochondrial biogenesis, circadian medicine represents exciting opportunities for translational therapy in intensive care, which is currently lacking. Circadian medicine also represents a link to metabolic determinants of outcome, such as diabetes and cardiovascular disease. More than ever, we are appreciating the problem of circadian desynchrony in intensive care. This review explores the rationale and evidence for the importance of the circadian clock in surviving critical illness.

*The only reason for time is so that everything doesn’t happen at once.*

—Albert Einstein

The regular 24-h environmental cycle generated by the planet’s rotation has driven the evolution of intrinsic biological timing mechanisms in virtually all life forms on the Earth. In mammals, circadian clocks orchestrate daily rhythms in biology and behavior, such that most physiological systems are regulated in a time-of-day-dependent manner (Dibner et al., 2010; West and Bechtold, 2015; Bass and Lazar, 2016). Please see Figure 1. for summary of pertinent critical care physiology regulated by circadian ‘clock’ genes.

**Figure 1.**
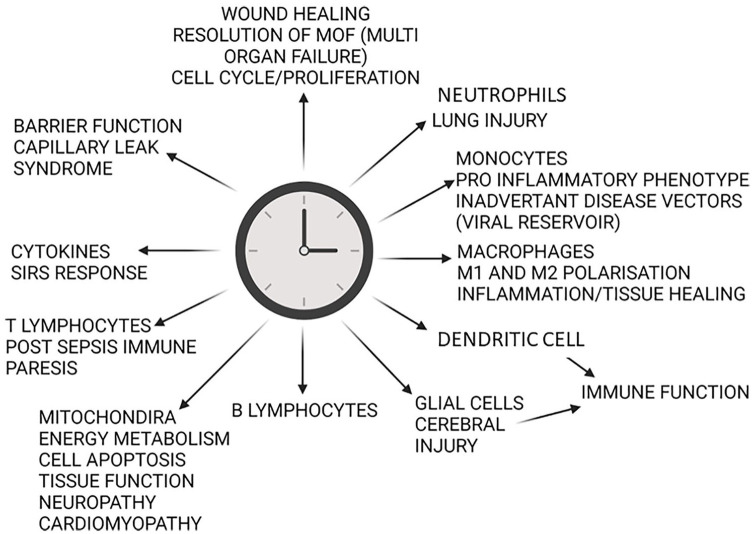
Summary of points of control exerted by the circadian clock relevant to patient physiology in intensive care.

The core cellular circadian pacemaker in mammals oscillates with a 24-h period and is driven by a transcriptional-translational negative feedback loop, with positive transcriptional regulators BMAL1/CLOCK promoting expression of repressors *Period (Per1/2), Cryptochrome (Cry1/2), Nr1d1*, and *Nr1d2* (REV-ERBα/β) (King et al., 1997; Becker-Weimann et al., 2004; Brown and Doyle, 2020) and activators such as RORs (RORa, RORb, and RORys). The core clock transcription factors act through clock-controlled genes to regulate multiple aspects of cellular function in a cell- and tissue-specific manner.

Both energy metabolism and immunity are strongly regulated by the circadian clock, shaping physiological and immune responses based on the time of day. Because of the pervasive nature of the clock within our biology, altered circadian rhythmicity has been recognized as a characteristic feature and/or contributing factor to numerous diseases states, with clear implications for diagnosis and treatment (Allada and Bass, 2021).

The circadian clockwork regulates all major cell types and pathways in the immune system, from differentiation, trafficking, and local cell-based responses (Scheiermann et al., 2018). Our group was the first to define the role of specific clock components in shaping immune and inflammatory responses to challenges (Gibbs et al., 2012, 2014; Kitchen et al., 2020) and how inflammatory signals acutely re-set circadian circuits via the rapid selective degradation of specific core proteins of the circadian clockwork (Pariollaud et al., 2018). In addition, energy metabolism is profoundly affected by timed feeding and fasting events, and also by the internal circadian machinery, affecting lipogenic and lipolytic cycles in the liver, and adipose, as one example (Hunter et al., 2020). In the context of critical illness, the influence of the endogenous circadian clock, plus the altered, and typically circadian disruptive, intensive care environment act together to drive physiology and pathophysiological responses. Here, we review why targeting circadian clock genes, for example, with the REVERB agonist SR9009 and others, may be of benefit for patients in intensive care.

In the context of the stress imposed by critical illness, there are some additional considerations. These include the role of the circadian clock in driving cycles of mitochondrial fission and fusion, thereby determining the cellular bioenergetic status. Here, the cellular clock drives a circuit involving the mitochondrial master regulator PGC1a, through REVERBa (L. Sun et al., 2021). In addition, inflammation-driven HIF1a stabilization may affect circadian function through heterodimerization with the core clock protein BMAL1; HIF1, and BMAL1 are homologous as bHLH-PAS domain proteins (Peek et al., 2017; Adamovich et al., 2017). These connections suggest that critical illness may provide a rather specific cellular environment to re-wire circadian regulatory networks, with therapeutic implications.

## Intensive Care Environment

Intensive care is characterized by high nursing ratios and active support of organ failure using artificial scaffolds of tissue function—for example, ventilators are used to facilitate gas exchange and blood filtration supports the poor renal function.

Whatever the reason for intensive care admissions, such as infection, major surgery, or trauma, admissions are typically complicated by ongoing inflammation, lack of homeostasis, metabolic derangement (Singer, 2017), and repeated nosocomial infection secondary to an acquired immune dysfunction (Ward et al., 2008). The most basic cell functions—such as that of ATP generation (Singer, 2017) and pH regulation—are lost (Al-Jaghbeer and Kellum, 2015), necessitating artificial intervention, until such time normal tissue and cell function is resumed.

Throughout intensive care admission, the initial often hyperinflammatory human phenotype in sepsis or trauma is followed by immune paresis (Nakamori et al., 2021), classically mediated by programmed cell death of T cells via the programmed death ligand pathway (PD1), which has been extensively described in COVID-19 patients (Venet et al., 2021). Although there are obvious ways to suppress inflammatory overactivity, such as the use of steroids, these do less for the immune paresis that often follows infection (Nakamori et al., 2021). However, there are many ways to modulate immunity and the body uses them in vivo—one such pathway is regulation, and especially suppression, of cellular circadian clock proteins (Coiffard et al., 2019). It has been shown in human patients in the intensive care unit that initial inflammatory insults—for example, bacterial infection—cause an initial loss in the expression of clock-related genes (Lachmann et al., 2021) in circulating immune cells. One mechanism for this is described below.

Alarmins and antigens, such as lipopolysaccharide (LPS), cause a profound suppression of clock proteins, for example, BMAL1 (Curtis et al., 2015; Diaz et al., 2020). This is mediated partly via upregulation of miRNA-155 (Curtis et al., 2015)—a small noncoding RNA produced in response to DAMPS (damage-associated molecular patterns), alarmins (molecules that signal cell “danger”), LPS, IL-6, and other toxins. This miRNA is well known to potentiate macrophage function and acts mechanistically to stabilize TNFa (Bala et al., 2011). However, it seems that human and murine BMAL1 transcripts both have binding sites for miRNA-155 and are inhibited by it (Curtis et al., 2015). Thus, it seems an evolutionary adaptation to suppress BMAL1, and thereby other clock genes such as CRY and PER in the setting of cell stress—as it is seen in immune cells of septic (Lachmann et al., 2021) as well as heavily injured trauma patients (Coiffard et al., 2019). In mice, experimental BMAL1 knockout provides a survival advantage in streptococcal pneumonia infection (Kitchen et al., 2020), mediated by improved phagocytic activity and immune cell recruitment (Kitchen et al., 2020), providing experimental evidence that the regulation of clock components has an effect on sepsis responses.

Urinary and serum melatonin and its precursors or metabolites have grossly abnormal circadian oscillations in human patients admitted to intensive care (Oldham et al., 2016). Furthermore, physiological parameters such as core body temperature and the “normal” circadian architecture of blood pressure variation are shown to be disturbed in multiple analyses of human intensive care patients (Chan et al., 2012).

It is not surprising that in very sick individuals, circadian rhythms are disturbed. However, recovery of externally measured circadian factors such as body temperature, heart and respiratory rate, and blood pressure appears to coincide with recovery from the disease state itself (Davidson et al., 2021). What is less clear, as there are no longitudinal studies of circadian biomarkers or gene transcripts throughout human intensive care stays, is when the cell clock resumes normality, and whether this affects disease trajectory. So far there is just evidence that on admission to intensive care, clock gene transcripts are much reduced in expression, and disordered in rhythm (Coiffard et al., 2019; Lachmann et al., 2021; Lazreg et al., 2013) at least in sepsis and trauma. One study shows that in neurologically injured patients (who may therefore have damage to the suprachiasmatic nucleus and other parts of the circadian system), clock genes are impaired for at least 1 week after admission (Diaz et al., 2020). What we do not know is whether clock gene regulation remains disordered at discharge.

Investigation is complex, because the effects of the cell clock are often tissue-specific, and many of the output pathways are affected by the state of the patient and confounded by the environment. For example, melatonin production from the pineal gland is regulated both by the central clock in the suprachiasmatic nucleus, but also suppressed by light, and so ambient lighting in the intensive care unit can affect melatonin measurements.

Light-dark therapy has thus far had extremely limited success in RCTs (randomised control trials), and this disappointment probably represents the extremely deranged inner milieu of physiology and biochemistry in extremis, thus will not be discussed in detail. Several trials have been done and do not appear to reduce important outcomes like delirium (Simons et al., 2016; Smonig et al., 2019). It is also worth noting other environmental cues to circadian entrainments, such as feeding duration, in intensive care are often continuous (Stroud et al., 2003) rather than episodic, and thus anticipated to blunt circadian entrainment with the environment.

## Circadian Disruption in Sepsis

Sepsis is defined as a dysregulated immune response to infection (Singer et al., 2016). It can lead to multiorgan failure (MOF) and a requirement for organ support. Mortality is as high as 50% (Bauer et al., 2020).

One of the features of septic shock is a high cytokine burden (Osuchowski et al., 2006); predictive of early mortality (Remick et al., 2005). Two such mediators, which also serve as useful biomarkers, are IL-6 (Song et al., 2019) and TNFa (Leon et al., 1998; Osuchowski et al., 2006). Other influential cytokines in this setting are IL-1b (Danielski et al., 2020) (produced by the NLRP3 inflammasome), IFNγ (Miles et al., 1994), and IL-18 (Mierzchala-Pasierb et al., 2019).

The cytokine storm can be followed by immunosuppression in the form of a “compensatory anti-inflammatory response” (Ward et al., 2008) coined as CARS (Osuchowski et al., 2006), predisposing to nosocomial infection. There is evidence these proinflammatory cytokines are targets for circadian clock proteins; for example, REVERB represses TNFa expression levels in mice (Lewis et al., 2018), and IL6 has been regarded as the most circadian cytokines, under REVERB control (Gibbs et al., 2012).

With respect to CARS—the immunosuppressive phase of sepsis, one pathway is mediated by TNFa (Zheng et al., 1995), and another by PD-1 (programmed death ligand), reviewed recently for immunotherapy (Liu and Li, 2017). Both of these cause T-cell death, contributing to immune dysfunction (Liu and Li, 2017). There is now evidence from preclinical animal models that the circadian clock plays a role in the “checkpoint” regulating the PD1 (Deng et al., 2018). In this animal model (Deng et al., 2018), BMAL1 deficiency increased lethality from sepsis; this was mediated by higher levels of lactate-induced PD-1 expression, which was linked to T-cell apoptosis and MOF. Lethality was abrogated in the presence of anti-PD-1 antibodies (Deng et al., 2018). Furthermore, lactate has emerged as a useful biomarker for the severity of septic shock (Singer et al., 2016; Lee and An, 2016). Also intriguingly in the above study (Deng et al., 2018) was the demonstration that BMAL1 deficiency allowed increased PKM-2 glycolytic flux, leading to higher lactate. In the same study, inhibition of PKM-2 also reduced mortality and reduced PD-1 expression. Moreover, PKM-2-induced glycolysis has been shown to directly affect the NLRP-3 inflammasome (Xie et al., 2016), which has been reviewed here (Danielski et al., 2020) as a driver of sepsis outcomes, further strengthening the mechanistic links.

Thus, the clock has a role to play in both reducing proinflammatory cytokines and reducing immune suppression in sepsis. This fits the trend noted in animals and human patients, that there is early and late death in sepsis (Xiao et al., 2006; Daviaud et al., 2015), either from proinflammatory MOF or nosocomial superinfection (Daviaud et al., 2015).

There are other interesting circadian features in sepsis; as early as 1960, Halberg et al (1960) appreciated a time-of-day-dependent lethality to inhaled endotoxin. More recently, the Ray group showed that a mouse model BMAL1 knockout (Kitchen et al., 2020) had improved mortality in *Streptococcal pneumonia*, secondary to improved macrophage motility and phagocytic function. Since then, a further mouse model has demonstrated that there is a time-of-day-responsive lethality to septic insult (Lang et al., 2019), irrespective of the myeloid clock being present, and mortality increased three times if kept in constant dark. This shows that there is more than one regulatory cue affecting this time-of-day response. Constant dark appears to be a mammalian cue for hibernation and stupor (Lee, 2007), promoting enzymes involved in the metabolism of fat (Zhang et al., 2006).

This would appear to be an unhelpful adaptation in the acute stages of sepsis, where it has been shown to increase the lethality of sepsis in a mouse model (Geiger et al., 2021), perhaps as early responses are dominated by a glucose requirement, rather than the mobilization of lipids.

In summary, it would therefore appear the interplay of light, feeding, myeloid clocks, and inflammation is complicated and requires further elucidation. The use of clock-related pathways as targets appears promising in animal models and requires human phase 1 safety trials.

## Circadian Disruption in Vascular Disease

Cardiovascular events are a common source of admission and cause of death in intensive care (Cook et al., 2021). Post cardiac arrest care takes place in the unit, and a period of cardiac “stunning” is common (Laurent et al., 2002). A comprehensive review of the pathophysiology of postresuscitation syndrome is here (Neumar et al., 2008), including ischemia-reperfusion mechanisms, metabolic reprogramming, and autonomic/adrenergic remodeling.

What we see in animal experiments of clock gene manipulation following cardiac arrest is that the presence of an intact clock process is protective in the setting of cardiac ischemia (Hu et al., 2019)—BMAL1 is integral to the healthy fusion-fission maintenance of mitochondria (E. Li et al., 2020). Circadian clock gene knockout models predispose to dilated cardiomyopathy (Ingle et al., 2015) and accelerated age-related dilated cardiomyopathy (Young et al., 2014), not dissimilar to the cardiomyopathy seen in other mitochondrial sources of cardiac pathology such as chemotherapy-induced, in which doxorubicin (Chatterjee et al., 2010) causes changes and damages mitochondria (Yin et al., 2018), or hereditary (Friedreich’s ataxia) cardiomyopathy (Hanson et al., 2019), a disease where mitochondria are directly damaged by dysfunctional frataxin and subject to enhanced oxidative stress.

Furthermore, there is a significant circadian association to autonomic regulation of cardiac rhythm, with sympathetic input shown to increase the morning risk of ventricular fibrillation (Hayter et al., 2021). Temporal changes in autonomic output are also responsible for a time-of-day susceptibility to QT-prolonging drugs such as the antibiotic levofloxacin (Kervezee et al., 2016).

It has also been shown in vivo that BMAL1 knockout significantly increases micro and macrovascular risks by altering endothelial function and intimal hyperplasia (Bhatwadekar et al., 2017). The phenotype seen is described as a mimic of diabetic vascular changes. Plaque remodeling in coronary artery disease was also recently shown to be influenced by BMAL1 activity (Zhu et al., 2018). In coronary artery disease, the ROS protective effects of active BMAL1 conveyed protection. This is in contrast to the beneficial effects seen with BMAL1 loss in *Pneumococcal pneumonia*, where the absence of BMAL1 allows augmented inflammatory action.

There has been previous interest in time-of-day-related outcomes in cardiac surgery, noted in aortic valve replacement in human patients (Montaigne et al., 2018). Here, afternoon surgery improved both postoperative troponin release and other clinical outcomes. This does match the circadian pattern of inflammation in humans (peaking in the morning) (Smolensky et al., 2015). A meta-analysis (Fudulu et al., 2021) in several thousands of patients failed to replicate this finding. However, it is worth noting that outcomes already vary by the individual center (Soppa et al., 2019) (the aortic valve study was in a single center), and also that an arbitrary afternoon cutoff of 12:00 may not capture the full circadian range of response. With respect to translational approaches, the REVERB agonist SR9009 when given early after ischemia, in mice, improved ischemia-perfusion-related remodeling and cardiac function (Reitz et al., 2019). In addition to REVERB agonists, attention is currently being applied to the development of RORa ligands for cardiovascular disease, for example, they are overexpressed in acute myocardial infarction (Meng et al., 2021).

## Clock Genes as Targets for Lung Injury

The lungs are a key tissue supported in intensive care; in the United Kingdom, invasive ventilation can only occur in the intensive care unit. Inflammation in the lungs is circadian (Nosal et al., 2020), as has been noted particularly in asthma (Durrington et al., 2014) and also in physiological circadian variation in myeloid trafficking (Druzd et al., 2014).

Ventilator-induced lung injury (Dreyfuss and Saumon, 1998) is a phenomenon in which invasive ventilation directly damages the lungs, irrespective of the underlying condition. There are few epidemiological studies as it is difficult to differentiate from the primary condition (such as pneumonia or acute respiratory distress syndrome); however, in a study of 332 patients, 24% were seen to develop lung injury in the first 5 days of admission (Gajic et al., 2004). Ventilator-associated lung injury (VALI) is known to be a cause of iatrogenic mortality because in a landmark RCT of the lung-protective ventilation strategy, mortality was reduced significantly in those with low tidal volumes (Acute Respiratory Distress Syndrome Network et al., 2000). Later studies have confirmed lower tidal volumes are associated with lower levels of cytokine release (Parsons et al., 2005), such as TNFa (Chiumello et al., 1999) or IL-1b (Dolinay et al., 2012). VALI consists of pressure (Kolobow et al., 1987), volume (Carlton et al., 1990), or hyperoxia (Helmerhorst et al., 2017)-induced trauma to the integrity of lung architecture (Ware, 2013) leading to edema (Carlton et al., 1990), tissue destruction (Ware, 2013), and thus impaired gas exchange. The release of alarmins and cytokines causes systemic inflammation (Parsons et al., 2005) and contributes to MOF (Jaecklin et al., 2010; Chiumello et al., 1999; Plötz et al., 2004). Strategies to reduce VALI are therefore of great interest. Some of the aforementioned pathways that mediate ventilation-induced lung damage have been shown to be affected by clock gene agonists such as REVERB agonists, for example, the NLRP3 inflammasome (Wang et al., 2018). Targeting REVERB may be an option to combat the inflammasome activation seen as part of ventilation-induced lung injury (Liu et al., 2019; Dolinay et al., 2012).

Furthermore, TNFa is implicated in VALI with evidence that the blockade of TNFa is protective (Proudfoot et al., 2018; Bertok et al., 2012). A phase II human trial of anti-TNFa was stopped due to being underpowered (Ryan et al., 2020); however, this was recent, and so far no further human trials have been published. TNFa mediates its actions mainly through the NFkB pathway (Liu et al., 2000; Zhang et al., 2017), and REVERB agonists have been shown to target this intracellular cascade, for example by binding to p65 at its promoter in macrophages (Wang et al., 2018). In addition, REVERB agonists were found to improve mortality and reduce cytokine levels in nonalcoholic steatohepatitis, a disease process with prominent NFkB action (Griffett et al., 2020).

Hyperoxia can drive lung damage, an effect mediated by activation of the JNK/ERK-3 pathway (Li et al., 2007). This pathway can be targeted by REVERB, by transcriptional repression through the NCoR-HDAC3 repressor complex (Zhang et al., 2002; Yin and Lazar, 2005).

A late effect of ventilator-induced lung injury and acute respiratory distress syndrome (ARDS) is pulmonary fibrosis (Albert et al., 2019; Marshall et al., 1998; Cabrera-Benítez et al., 2012, 2014). Mechanical trauma to lung tissue resulting from forced ventilation in mice results in the epithelial-to-mesenchymal transition, through the actions of TGF (Cabrera-Benítez et al., 2012). Fibroblast changes cause increased collagen deposition (Tsukui et al., 2020). Fibrosis directly contributes to mortality (Cabrera-Benítez et al., 2014). REVERB agonists have been shown to protect against fibrosis in an animal model of lung fibrosis (Cunningham et al., 2020). They are also protective with respect to the epithelial-to-mesenchymal transition (Wang et al., 2021), as an inflammatory response, and reduce morbidity, and mortality in an animal model of smoke-induced lung damage (Wang et al., 2021).

Further underlining the role of the clock genes in lung injury, murine experiments demonstrate that the severity of VALI is gated by BMAL1—high tidal volumes are less traumatic in a BMAL1 knockout (Felten et al., 2018) mouse. The loss of BMAL1 was associated with less neutrophil ROS production in response to ventilator injury.

In a rat model, the REVERB agonist SR9009 rescued many of the deleterious changes induced by high ventilator tidal volumes (Li et al., 2014). The tidal volumes used in these experiments were vastly outside the clinical range, 10-40 mL/kg—in humans the “safe” range is 6-8 mL/kg (Acute Respiratory Distress Syndrome Network et al., 2000), so it is impressive to see this degree of reduction. Corresponding parameters include reduced leukocyte egress and reduced TNFa (Li et al., 2014).

Furthermore, tissue susceptibility to stress in the lung has circadian regulation according to a study demonstrating that the oxidative stress response pathways NRF-2/glutathione affected the degree of lung fibrosis developed in response to bleomycin (Cunningham et al., 2020). The authors found that the NRF-2 pathway caused the altered redox state/glutathione reserves that affected the time-of-day response to bleomycin. BMAL1 is shown to bind to an important E-box on the NRF-2 gene (Q. Sun et al., 2021). BMAL1, together with NRF-2, is a key coordinator of oxidative responses in cells (Chhunchha et al., 2020).

Another factor in indirect injury in invasive ventilation is the loss of intercostal and diaphragmatic muscle mass, which affects weaning from the ventilator (Nakanishi et al., 2019) and may reflect the wider myopathy that occurs in the critically ill (van Gassel et al., 2020). REVERB agonists can affect skeletal muscle oxidative and functional capacity (Woldt et al., 2013; Amador et al., 2018) and so may find an application in this context. Indeed, REVERB doping has been proposed in elite sports (Davies et al., 2019). REVERB may prevent muscle atrophy, so preserving muscle mass (Mayeuf-Louchart et al., 2017). This is an intriguing prospect for intensive care patients who suffer from profound muscle wasting (Koukourikos et al., 2014), despite calorie-calculated feeding (Stroud et al., 2003). Moreover, muscle biopsies in critically ill patients demonstrate “bioenergetic failure” in the sense there is a loss in ATP concentrations that correlates with the degree of protein loss (Puthucheary et al., 2018). This is a very important area in intensive care because in addition to ventilator weaning (Nakanishi et al., 2019), it affects survivors postdischarge (Owen et al., 2019).

## Clock Genes as Targets in Subarachnoid Hemorrhage

Circadian pathways influence the extent of brain damage in subarachnoid hemorrhage. They also predict recovery (Davidson et al., 2020). Furthermore, changes in circadian rhythm have been shown in neurocritical care to predict the development of an intracranial pressure spike 24 h in advance (Nogueira et al., 2017). In addition, both cryptochromes (Nogueira et al., 2017) and heme oxygenase directly influence the degree of inflammation and neuronal survival in subarachnoid hemorrhage (Li et al., 2018). Heme oxygenase is mentioned given it is a key enzyme in the degradation of heme, is increased locally in hemorrhagic situations as erythrocytes leave the intravascular space, and is also circadian (Li et al., 2018).

Subarachnoid hemorrhage remains a desperate condition; one-third of patients die within 3 months, while over half never fully recover (Andersen et al., 2019). Management is mainly limited to preventing further bleeding via intravascular coiling or neurosurgical clipping (Highton and Smith, 2013), the use of a calcium channel antagonist to limit vasospasm and further ischemia (Pickard et al., 1989) and supportive care (Highton and Smith, 2013).

Following the initial bleed, calcium channel antagonists are used to reduce further cerebral ischemia (Pickard et al., 1989)—defined as areas of cerebral infarction within 6 weeks of the initial bleed but not related to it directly (Vergouwen et al., 2010). There appears to be more than one mechanism of damage, for example, microthrombosis (Suzuki et al., 1990) in the symptomatic area, evident at autopsy in human patients, and endothelial dysfunction (Frijns et al., 2006), as well as toll-like receptor activation, which predicts the outcome in human patients (C. Ma et al., 2015), inflammasome activation in murine models (Greenhalgh et al., 2012), neuronal and glial toxicity in cell culture and animal models (Regan and Panter, 1993; Rosen and Frumin, 1979), and impaired mitochondrial function/mitophagy (Youn et al., 2021) evident in human CSF (cerebrospinal fluid) samples.

Following subarachnoid hemorrhage, one of the issues central to delayed cerebral ischemia is toxicity from hemoglobin (Suzuki et al., 2003); under physiological conditions, it produces free radicals (Reeder et al., 2004) that oxidize and damage cell lipids, proteins, and DNA (Marnett et al., 2003). Moreover, it induces a form of cell death in neurons called ferroptosis (Bai et al., 2020), only recently described (J. Li et al., 2020), and free heme that is toxic to mitochondria (Mercer et al., 2011).

The extent of bleeding in subarachnoid hemorrhage predicts mortality (van der Steen et al., 2020). In cell culture, heme directly induces cell death (Regan and Panter, 1993), recently confirmed as neuronal ferroptosis (Bai et al., 2020). Inhibition of ferroptosis reduces mortality in an animal model of subarachnoid hemorrhage (Li et al., 2017). Injection of blood, or heme, into the subarachnoid space evokes focal epileptogenesis and symptoms of delayed cerebral ischemia (DCI) in animal models (Rosen and Frumin, 1979). Moreover, chelation of heme reduces DCI in rabbits (Arthur et al., 1997) and primates (Horky et al., 1998), and higher levels of CSF ferritin (an endogenous iron-binding protein) are protective against DCI in human patients (Suzuki et al., 2003). Interestingly, haptoglobin genotype affected functional outcomes in humans (Kantor et al., 2014) after subarachnoid hemorrhage (Kantor et al., 2014)—haptoglobins provide important CSF heme scavenging (Galea et al., 2012). Chelation therapy failed in a phase II human trial for the main endpoint (outcome at 3 months) but was shown to be safe. One issue in the trial was that just 30% of patients received therapy within 12 h—where the animal benefit had been seen to depend on an early window of administration. One issue in patients is time-to-treatment is delayed by diagnosis and presentation (when patients seek medical attention/or are found), time to scan, scan reporting, appropriate referral, surgical intervention, and availability of research personnel for recruitment and administration of drug/placebo, yet cellular damage may occur before this intervention can take place.

REVERB agonists have been shown to have protective effects on ferroptotic injury (Guo et al., 2021). Furthermore, they can assist in improved mitochondrial functional capacity (Woldt et al., 2013) and turnover (L. Sun et al., 2021). Therefore, targeting REVERB may prove an attractive option, in the event that higher potency, and more specific agents emerge from drug discovery programs. There are very few reports of REVERB therapies in neurological preclinical or clinical studies; yet, recent interest in the circadian regulation of disease is taking shape. Stroke infarct size has significant circadian variability and is now under great scrutiny for improving outcomes and secondary prevention of injury (Lo et al., 2021).

Clock pathways therefore represent a truly exciting prospect for the management of critical care neurological conditions. Pathways affected by clock genes are often redox/mitochondrial-related, unsurprising given their origin as phase mediators of UV light toxicity and energy metabolism.

Some impressive studies now suggest that the failure of translation of animal-to-human neuroprotective therapies is an inevitable consequence of administering the intervention to people at the wrong circadian phase, thereby leading to failure in clinical studies (Esposito et al., 2020).

## Clock Genes as Targets in Acute Kidney Injury

Acute kidney injury (AKI) is a spectrum of renal disease defined by standardized criteria, based on creatinine changes and/or urine output (Makris and Spanou, 2016). Mortality in intensive care is high, exceeding 50% (Pickkers et al., 2021). In addition to this, organ support with renal replacement therapy is expensive, costing hundreds to thousands of dollars a day (Srisawat et al., 2010). It requires 1:1 nursing and is therefore labor-intensive. It also requires invasive venous access, which risks infection (Atilla et al., 2016).

A recent review encapsulates the pathophysiology of AKI (Makris and Spanou, 2016). Of particular importance is the activation of immune receptors on the glomerular endothelium (Radi, 2018). This leads to local inflammation and also microthrombosis, the occlusion of capillaries by small clots (Chang, 2018)—which reduces oxygen supply to tissues; renal tissue hypoxia is associated with AKI and can be measured directly in animals (Iguchi et al., 2019) and noninvasively in human patients. (Silverton et al., 2021)

In addition to these observations, metabolic deficits have been seen in AKI, for example, abnormal fission and fusion of mitochondria (Brooks et al., 2009; Yan et al., 2020). In particular, the abnormal fission may be driven by dynamin-related protein 1 (DRP-1) (Perry et al., 2018). Mitochondrial biogenesis is driven by PGC-1a (Goffart and Wiesner, 2003; Jornayvaz and Shulman, 2010), and PGC-1a dysregulation is seen in models of AKI development (Fontecha-Barriuso et al., 2020), with increased expression improving outcomes (Tran et al., 2011). Similarly, NAD+ deficiency is seen in AKI (Poyan Mehr et al., 2018). Supplementation of NAD+ is successful in improving creatinine levels in human phase 1 trials (Poyan Mehr et al., 2018). The protective effect of NAD+ on mitochondrial function is mediated by Sirtuin 3 (Lombard et al., 2011; Fan et al., 2021).

Because the clock gene REVERB can affect mitochondrial dynamics via the NRF-2 pathway (Goffart and Wiesner, 2003; Eichenfield et al., 2016; Li et al., 2021; L. Sun et al., 2021). It may be an attractive target either for renal protection (prior to a planned insult such as major surgery [Prowle et al., 2021], or contrast dye [Lei et al., 2018]) or for reducing the severity of AKI in intensive care. REVERB agonists increase mitochondrial oxidative capacity in skeletal muscle, but their role in the kidney has not been explored.

Therefore, this is a translational opportunity for a condition that currently requires time and expensive organ support where there are no published studies on REVERB agonists in the context of sepsis-induced AKI, or to reduce perioperative AKI.

## Circadian Disruption and Immunometabolism

**Figure 2.**
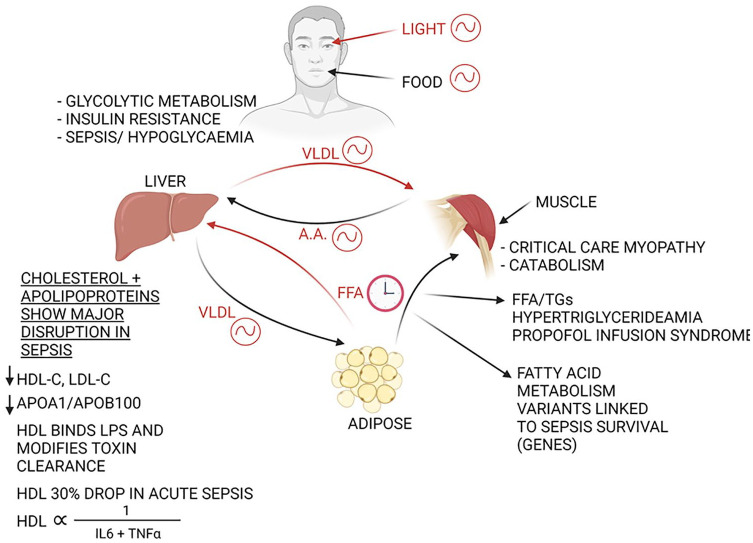
Summary of circadian points of control over energy metabolism relevant to intensive care. The disruption to light and food entrainment mechanisms is central to the disruption in physiology seen. The precise coordination or lipid metabolism between the liver, muscle, and adipose, which is essential for health adaptation to rest and active periods, is both clock-regulated and also subject to direct regulation by inflammatory signaling in critical illness. Abbreviation: LPS = lipopolysaccharide FFA = Free Fatty Acid; TGs = Triglycerides; VLDL = Very Low Density Lipoprotein; HDL-C = High Density Lipoprotein Cholesterol; LDL-C = Low Density Lipoprotein Cholesterol.

For a summary of the metabolic pathways regulated by the circadian clock, please see Figure 2. Early in the systemic inflammatory response, there is a “glycolytic switch”—seen in initially activated neutrophils (Sadiku et al., 2021), macrophages (Yu et al., 2020), B cells (Doughty et al., 2006), proinflammatory T cells (Chapman et al., 2020), and microglia (Cheng et al., 2021). Therefore, metabolic regulation of inflammation is gaining traction (Soto-Heredero et al., 2020). Inhibiting glycolytic changes appears to modify mortality in mice with septic shock—mediated via PMK-2 (Deng et al., 2018). This is logical when considering the effects of glycolysis on proliferation, cell activity, and the production of proinflammatory cytokines (Van Wyngene et al., 2018), which correlate with the severity of septic shock (Leon et al., 1998; Song et al., 2019). The liver transcriptome and proteome are profoundly regulated by the circadian clock (Droin et al., 2020; Weger et al., 2021), with BMAL1 driving changes in carbohydrate (Ma et al., 2016) and lipid metabolism (Zheng et al., 2016). Circadian disruption, such as that produced by shiftwork (Sharma et al., 2017) or continuous feeding (Hutchison et al., 2019) (ubiquitous in critical care), predisposes to impaired glucose tolerance. Glucose intolerance is a poor prognostic factor in sepsis (Bingham et al., 1980) and was noted as early as the 1970s (Bingham et al., 1980) to be associated with a state of hyperglucagonemia, reflecting a “metabolic energy deficit.” Hyperglucagonemia and high glucose cause inhibition of a number of intermediate cell pathways including sirtuins and PGC-1a (Vieira et al., 2013). PGC-1a and the related mitochondrial regulator genes are prognostic factors for the outcomes after intensive care admission (Huang et al., 2021), and are heavily involved in mitochondrial fission-fusion homeostasis (Jornayvaz and Shulman, 2010), critical for the regulation of oxidative phosphorylation relevant for sepsis survival (Singer, 2017; Nedel et al., 2021). Interestingly, inhibition of aerobic metabolism is protective against sepsis-induced AKI in some animal models (Tan et al., 2021).

One of the other key developments in critical care is the acknowledgment that immune paresis is seen in patients (Ward et al., 2008), leading to nosocomial secondary infections, such as ventilator-associated pneumonia, intravenous line infections, and opportunistic infection (Ward et al., 2008). This immune paresis is related to the loss of T lymphocyte function (Kühlhorn et al., 2013), with enhanced T-cell apoptosis (Monneret et al., 2016) (secondary to TNFa (Zheng et al., 1995) and increased levels of programmed death ligand—PD-1 (Liu and Li, 2017)]. Specific T lymphocyte subsets, such as Treg cells, have a special role in sepsis recovery—sepsis is a biphasic disease, with either early or late mortality (Nedeva et al., 2019).

Early Treg cell metabolism is glycolytic (Chapman et al., 2020)—the Warburg effect shunting substrate into advanced proliferation and cell division, with the GLUT 1 transporter prominently expressed (Macintyre et al., 2014). However, in the passage of days, metabolism begins to favor fatty acid/glutamine substrate oxidation, and differentiation of proresolution T lymphocytes (Kominsky et al., 2010; Arts et al., 2017).

This metabolic polarization matched to function is also reflected in the glycolytic inflammatory M1 macrophage phenotype and the fatty acid-favoring anti-inflammatory/tissue regenerating M2 macrophage (Arts et al., 2017). Closely coordinated with these metabolic needs is the acute suppression of mitochondrial respiration in illness (Singer, 2014; Carré et al., 2010; Singer, 2017).

Moreover, a small number of case reports (Wischmeyer and San-Millan, 2015) have shown that the recovery of fatty acid beta-oxidation after critical illness matches quality-of-life outcomes, such as the ability to exercise. Other studies show that muscle quality and mitochondrial deficits persist after intensive care which influences rehabilitation (Owen et al., 2019).

The importance of fatty acid metabolism in sepsis became important when a seminal paper identified that defects in fatty acid beta-oxidation in children dictated survival from sepsis in pediatric critical care (Wong et al., 2009). There, gene variations in PGC-1a, a master regulator of PPAR, fatty acid oxidation, and mitochondrial biogenesis (mitochondria and fatty acid oxidation are twinned) underpinned survival.

Immunometabolic work has shown that fatty acid beta-oxidation is especially important in promoting the M2 macrophage phenotype (Arts et al., 2017), encouraging wound healing and tissue regeneration. It is also the favored pathway in slightly older (i.e. T cells that are several days post-TCR activation) Treg cells (Kempkes et al., 2019), which have an anti-inflammatory function. REVERB has been shown to affect cellular fatty acid metabolism in muscle (Amador et al., 2018), but actions on energy substrate utilization in immune cells remain unexplored.

The metabolic and effector function changes underpin and explain the clinical picture seen in sepsis—acute hyperinflammation with early death (Daviaud et al., 2015)—excessive innate immunity (being the immune response that does not require pre-exposure, requiring neutrophils, macrophages, etc., versus adaptive immunity, which is an acquired response directed by B and T), or protracted immune paresis/inappropriate tolerance, nosocomial infection, and late death (Daviaud et al., 2015). Clearly, the circadian control of energy metabolism and thereby regulation of effector function of immune cells represents an exciting breeding ground for novel therapeutic targets.

Briefly, cholesterol metabolism also influences immunity (Sharma et al., 2019), for example, a dramatic reduction in HDL is seen in sepsis (Morin et al., 2015), with actions of bacterial products including LPS (Liu et al., 2015). Inhibition of a cholesterol transporter significantly improves sepsis survival (Trinder et al., 2021) and is under clock control (D. Ma et al., 2015).

## Conclusion

Spatiotemporal regulation of the transcriptome of the liver at least has been elegantly illustrated (Droin et al., 2020). The circadian clock helps provide a “metronome” for coordinating metabolism (Weger et al., 2021), hypoxic/oxidative stress (Cunningham et al., 2020; O’Connell et al., 2020), and immune function (Guo et al., 2019), to provide the best “setting” for response to external triggers (Lang et al., 2019).

Holistic coordination of the transcriptome at a 3D chromatin level has been missing from the management of diseases in intensive care. These patients have dysregulated immune systems (Osuchowski et al., 2006), mitochondrial failure (Singer, 2017), and aggressive muscle loss (Nakanishi et al., 2019). Immunomodulation in critical care has been controversial (Young and Marsh, 2018), with only recently steroids and Tocilizumab being licensed for COVID-19 (RECOVERY Collaborative Group et al., 2021; Abani et al., 2021). However, steroids are associated with muscle atrophy (Hasselgren et al., 2010). There are no currently licensed treatments to improve mitochondrial function in intensive care (Kozlov et al., 2011).

REVERB manipulation and therapeutic targeting of other clock-related pathways represent a truly ground-breaking opportunity to improve multiple axes of illness, and further studies in this area may clarify why current light-related trials have failed to succeed (Simons et al., 2016). Although REVERB targets have currently the most diverse usage in preclinical models, other clock-related gene targets exist. For example, CRY stabilizers prevent the degradation of CRY, and their use in *Drosophila* appears to be life-extending and beneficial under starvation conditions (Solovev et al., 2021), although there are limited publications regarding studies in inflammatory illness. ROR modulators appear to have more preclinical usage than CRY stabilizers at present, especially in relation to autoimmune disease. They have been recently reviewed elegantly (Ladurner et al., 2021). The RORs have different tissue expressions; RORa is present predominantly in organs, while RORy is lymphatic (Ladurner et al., 2021); these different isoforms may provide nuanced targeting of cellular pathways. RORa has proven a target in LPS-associated septic shock in mice (Hams et al., 2021), as well as surviving bacterial infection following severe burns injury, in rodents (Ito et al., 2018). RORs are heavily intertwined with lipid metabolism and homeostasis (Jetten et al., 2018), which has made them a focus for diabetes for example, although the immediate critical care implications are unclear. They have also been recently reviewed in the treatment of myopathies (Welch and Flaveny, 2017), which is highly pertinent to the catabolic, cachexic state of seriously unwell patients as mentioned throughout this review.

The unlicensed use of these drugs as performance enhancers (Davies et al., 2019) raises the issue of how to enhance “performance” in the critically ill. The early phase of clinical trials of new, circadian clock therapeutics is now urgently needed.
